# Extreme D-dimer Elevation and Hemodynamic Collapse Due to Splenic Rupture in an Anticoagulated Patient

**DOI:** 10.7759/cureus.98163

**Published:** 2025-11-30

**Authors:** Isa Alshamsan, Reem Alameri, Harikrishnan Nair

**Affiliations:** 1 Internal Medicine, Betsi Cadwaladr University Health Board, Rhyl, GBR; 2 Internal Medicine, Royal College of Surgeons in Ireland, Dublin, BHR; 3 Nephrology, Betsi Cadwaladr University Health Board, Rhyl, GBR

**Keywords:** anticoagulation, d-dimer, diagnostic anchoring, emergency medicine, hemoperitoneum, splenic rupture

## Abstract

D-dimer is commonly used in acute medicine to evaluate suspected venous thromboembolism (VTE), but markedly elevated levels lack specificity and may also occur in serious non-thrombotic conditions. We report a case of an 83-year-old woman on anticoagulation who presented with a two-week history of left-sided pleuritic chest pain and a profoundly elevated D-dimer (>10,000 ng/mL). Initial CT pulmonary angiography excluded pulmonary embolism, and she was admitted for observation and empirical antibiotics. Later the same day, a repeat D-dimer test showed an increase to >14,000 ng/mL. Overnight, she developed hypotension and a rapid hemoglobin drop from 119 to 77 g/L, accompanied by abdominal distension and tenderness. Urgent CT abdomen and pelvis revealed a large hemoperitoneum with suspected splenic rupture. Anticoagulation was reversed, and she underwent emergency laparotomy, confirming splenic rupture with successful hemostasis. Post-operative recovery was uncomplicated. Histology revealed an underlying vascular neoplasm, which may have contributed to splenic fragility but did not alter the acute diagnostic challenge. This case illustrates the difficulty of interpreting extreme D-dimer elevations and emphasizes that sudden hemodynamic deterioration in anticoagulated patients may indicate life-threatening hemorrhage rather than thromboembolism. Clinicians should maintain a broad differential diagnosis and repeat clinical assessment when initial investigations are unrevealing, ensuring timely recognition of rare but critical causes of collapse, such as splenic rupture.

## Introduction

D-dimer testing is integral to the diagnostic evaluation of suspected venous thromboembolism and is often used with validated clinical decision tools to safely exclude pulmonary embolism or deep vein thrombosis in low-risk patients [1]. However, D-dimer is highly sensitive but non-specific; markedly elevated values can occur in several conditions unrelated to thrombosis, including infection, malignancy, disseminated fibrinolysis, and major bleeding [2]. In the context of acute hemorrhage, activation of the coagulation cascade with subsequent secondary hyperfibrinolysis can produce extremely high D-dimer levels, complicating interpretation.

Several viral infections, most notably Epstein-Barr virus and cytomegalovirus, are recognized causes of atraumatic splenic rupture due to splenic congestion and capsular fragility [3-5]. Although varicella-zoster virus (VZV) is less well documented as a precipitant, recent infection may still contribute to splenic vulnerability. Splenic rupture can also occur in individuals receiving anticoagulation, even in the absence of trauma [6,7]. Awareness of the diagnostic biases introduced by extreme biomarker abnormalities is essential, particularly when initial imaging does not provide a clear explanation for the patient’s presentation.

## Case presentation

An 83-year-old woman presented with a two-week history of intermittent left-sided pleuritic chest pain. She denied trauma, dyspnea, syncope, or abdominal pain. Her history included paroxysmal atrial fibrillation and a recent shingles episode, although the dermatome distribution was not documented, limiting assessment of symptom correlation. Her medications included edoxaban and amlodipine.

**Figure 1:**
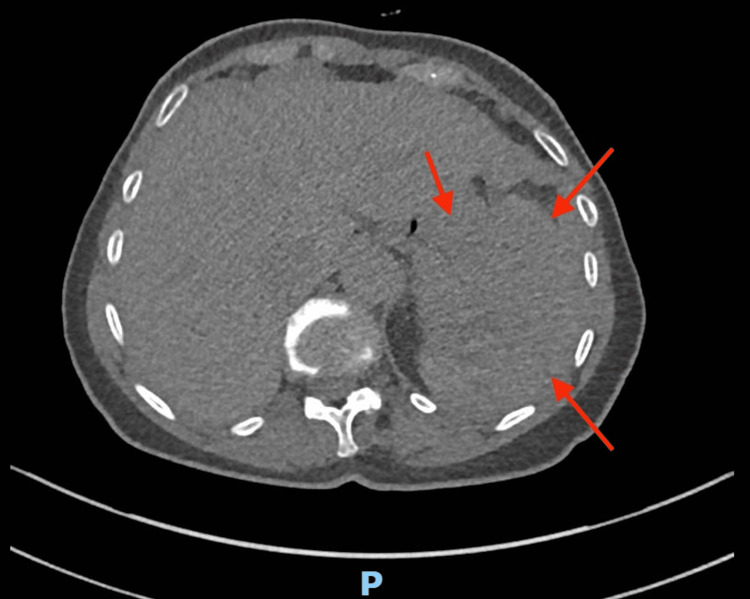
CT pulmonary angiography showing the upper pole of the spleen. Red arrows indicate areas reviewed for subtle contour irregularity on retrospective assessment. No definite abnormality was identified due to limited splenic coverage and non-splenic contrast timing.

On admission, she was hemodynamically stable. Initial laboratory studies showed a D-dimer of 10,000 ng/mL, normal hemoglobin, platelet count, inflammatory markers, and coagulation parameters. ECG confirmed atrial fibrillation. CT pulmonary angiography (CTPA) excluded pulmonary embolism, with only the upper pole of the spleen partially visualized (Figure 1).

Retrospective review of the CTPA revealed subtle contour irregularities at the splenic margin; however, the study was performed in a pulmonary arterial phase and lacked a dedicated splenic contrast phase. No definite subcapsular collection, perisplenic stranding, or free fluid was identified. Given the limited splenic coverage, early splenic pathology could neither be confirmed nor excluded.

**Figure 2:**
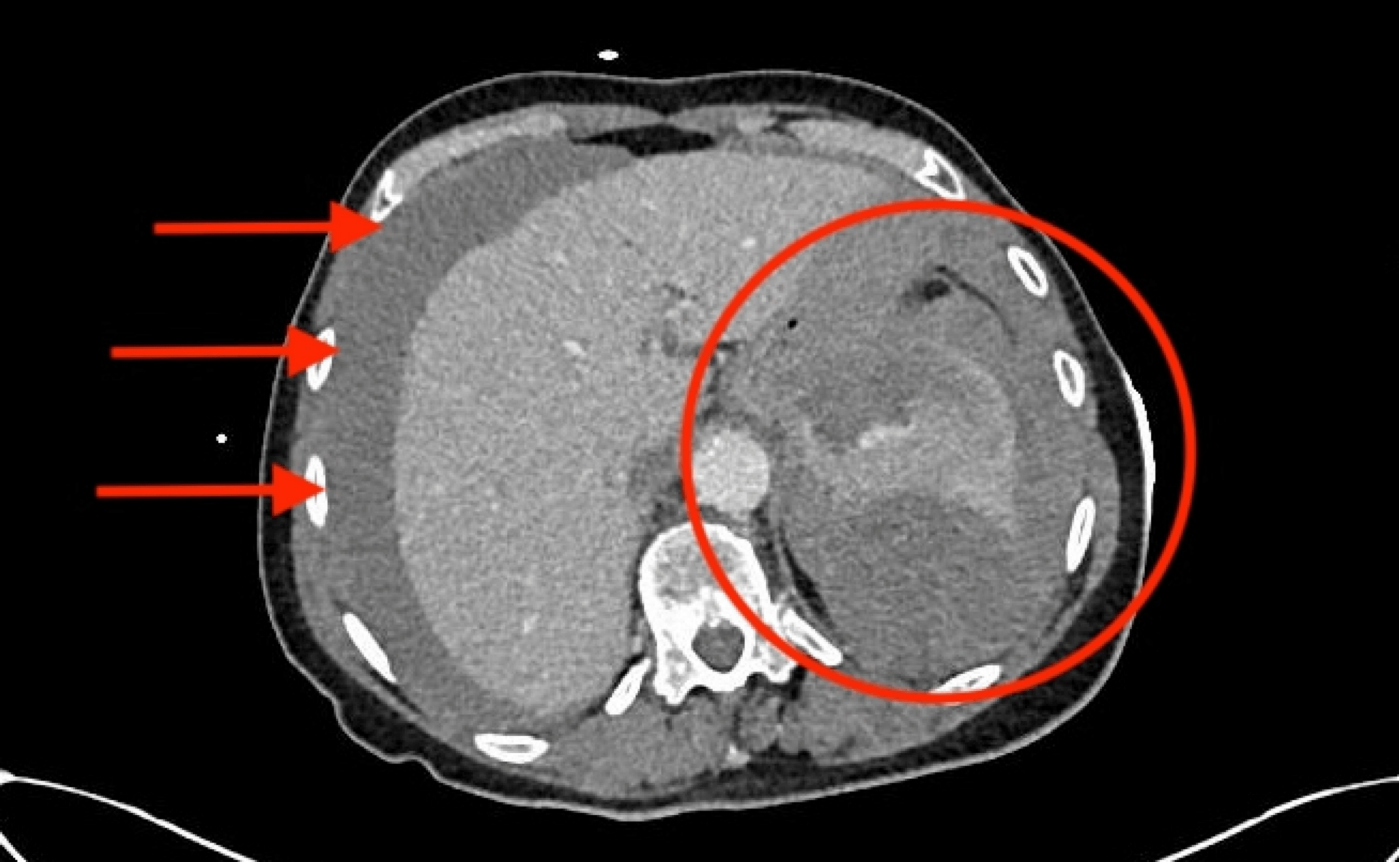
Axial CT abdomen/pelvis at a mid-splenic level showing hemoperitoneum and active splenic bleeding. Red arrows highlight contrast extravasation. Red circle denotes the main region of hemorrhage.

**Figure 3:**
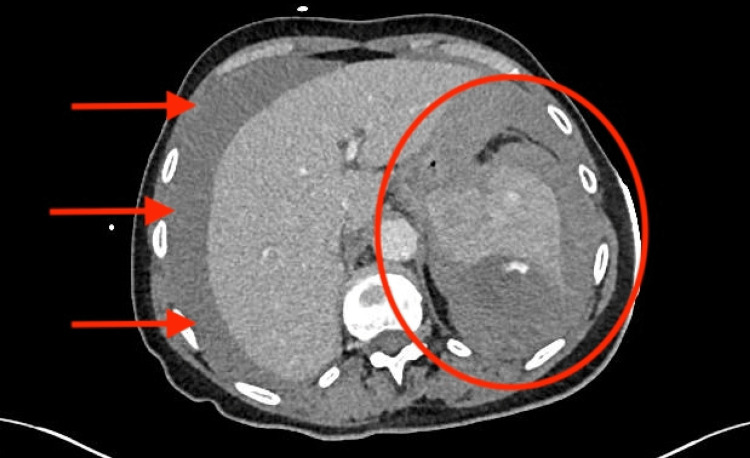
Axial CT abdomen/pelvis at a more inferior splenic level demonstrating the further extent of hemoperitoneum and dependent fluid collection. The red arrow highlights the contrast extravasation, and the red circle indicates the main region/site of hemorrhage.

She was admitted for observation. Later that day, a repeat D-dimer rose to 14,237 ng/mL. Overnight, she developed hypotension and new abdominal distension with a hemoglobin drop from 119 to 77 g/L (Table 1). She was resuscitated with intravenous fluids and blood products. Urgent CT abdomen/pelvis revealed a large hemoperitoneum with features of splenic rupture (Figures 2, 3).

**Table 1:** Serial laboratory values from presentation to diagnosis of splenic rupture. PT: prothrombin time; APTT: activated partial thromboplastin time

Time point	Hb (g/L)	PT (s)	APTT (s)	Fibrinogen (g/L)	D-dimer (ng/mL)	Platelets (×10⁹/L)
Day 1 (pre-collapse)	119	11	24.3	2.7	10,000	170
Day 1 (rising D-dimer)	-	-	-	-	14,237	-
Day 1 (post-collapse)	77	12.4	27	2.7	-	164
7 h post-collapse (post-transfusion)	126	11	24.7	2.7	-	77

**Figure 4:**
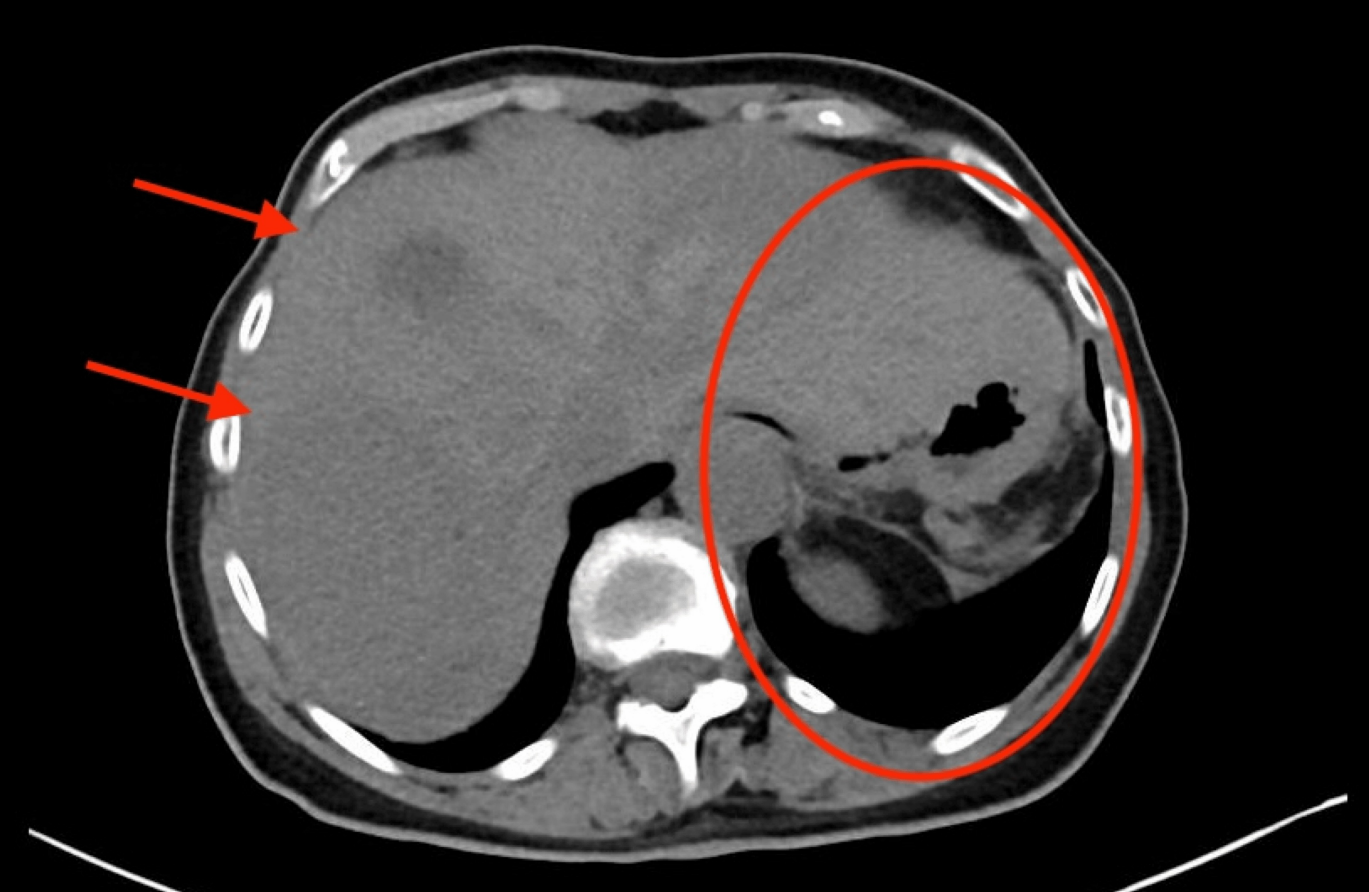
CT abdomen/pelvis post-splenectomy, showing evacuation of hemoperitoneum. Red arrows indicate post-operative changes. Red circle marks the splenectomy bed.

Given the severity of bleeding, edoxaban was reversed using prothrombin complex concentrate (PCC) in accordance with guideline recommendations for factor Xa inhibitor-associated major hemorrhage. She underwent emergency laparotomy and splenectomy, which confirmed active splenic bleeding (Figure 4).

Histopathology report showed that the spleen contained a circumscribed vascular neoplasm comprising spindle-shaped hypercellular cells with mild-to-moderate atypia arranged in nodules, with variably dilated vascular channels. Immunohistochemistry showed positivity for CD31 and CD34, confirming vascular differentiation; human herpesvirus 8 (HHV-8) and SOX-10 were negative. Mitoses were approximately 5 per 10 high-power fields, with no necrosis. The findings were consistent with a vascular neoplasm. Targeted sarcoma next-generation sequencing (NGS) demonstrated no gene fusion events. This lesion may have contributed to splenic fragility, but it did not change the acute management or diagnostic challenge posed by her presentation. Post-operative recovery was uneventful, and she was discharged in stable condition.

## Discussion

This case demonstrates how extreme D-dimer elevation can mislead clinicians toward thromboembolic causes despite the presence of life-threatening hemorrhage. In this patient, the initial D-dimer >10,000 ng/mL likely reflected early subcapsular splenic bleeding with activation of coagulation and secondary hyperfibrinolysis [2]. The subsequent rise to >14,000 ng/mL corresponded with the progression of hemorrhage, preceding clinical collapse.

The initial CT pulmonary angiography (CTPA) did not reveal a splenic abnormality; however, limited splenic coverage and arterial-phase timing restricted its sensitivity for detecting subtle capsular irregularity or subcapsular fluid. The rapid deterioration that followed supports the interpretation that hemorrhage was already occurring at the time of the initial scan.

Atraumatic splenic rupture occurs in a variety of contexts, including viral infections (particularly Epstein-Barr virus {EBV}, cytomegalovirus {CMV}), hematologic diseases, anticoagulant therapy, and neoplastic infiltration [3-7]. Varicella-zoster virus (VZV) is not well documented as a direct cause, and the absence of viral inclusions on histology makes any association in this case speculative. The vascular neoplasm identified on histology likely contributed to capsular fragility, classifying this as an atraumatic but pathological rupture rather than a true spontaneous rupture.

Anticoagulation may exacerbate blood loss once capsular disruption occurs, accelerating hemodynamic compromise. Diagnostic anchoring on extreme D-dimer values contributed to an initial focus on pulmonary embolism, demonstrating how reliance on biomarkers can obscure alternative life-threatening diagnoses.

## Conclusions

Extreme D-dimer elevation is not specific to thromboembolism and can be a marker of severe hemorrhage. In anticoagulated patients with evolving symptoms or hemodynamic instability, clinicians should promptly reassess, consider intra-abdominal bleeding, and proceed to urgent imaging when deterioration occurs. Maintaining a broad differential diagnosis helps ensure early recognition of rare but critical conditions such as splenic rupture.
